# The Confirmation of Safety for the Intensified Conditioning Regimens: A Retrospective Study of Allogeneic Hematopoietic Stem Cell Transplantation for Non-Remission Hematological Malignant Diseases 

**Published:** 2018-04-01

**Authors:** Shuro Yoshida, Hideho Henzan, Toshiyuki Ueno, Takuya Shimakawa, Yayoi Matsuo, Takuro Kuriyama, Noriyuki Saito, Ichiro Kawano, Akihiko Numata, Ken Takase, Tadafumi Iino, Tetsuya Eto

**Affiliations:** 1 Department of Hematology, Hamanomachi Hospital, Fukuoka, Japan; 2 Department of Hematology, Matsuyama Red Cross Hospital, Ehime, Japan; 3 Department of Medicine and Biosystemic Science, Kyushu University, Fukuoka, Japan; 4 Department of Hematology, National Hospital Organization Kyushu Medical Hospital, Fukuoka, Japan; 5 Hematology and Oncology Division, Japan Red Cross Fukuoka Hospital, Fukuoka, Japan

**Keywords:** Non-remission diseases, Intensified conditioning, Sequential and additional chemotherapy, Allogeneic hematopoietic stem cell transplantation

## Abstract

**Background:** The prognosis of allogeneic hematopoietic stem cell transplantation (HSCT) for non-remission hematological malignant diseases is usually unfavorable. The most uncontrollable factor is residual disease or relapse. To overcome this problem, intensified conditioning regimens- sequential and/or additional chemotherapy to the standard regimen- could be effective. However, increasing the intensity of conditioning might also lead to more complications.

**Materials and Methods:** We retrospectively analyzed 81 patients with non-remission disease who received allogeneic HSCT in our institution between 2007 and 2011.

**Results:** 55.6% in 36 myeloablative conditioning patients and 46.7% in 45 reduced-intensity conditioning patients received intensified conditioning. The 5-year probability of overall survival was 35.0% and 17.1% in the standard and intensified group, respectively (*p*=0.027). Relapse mortality was 30% in the standard regimen group and 36.6% in the intensified regimen group (*p*=0.54). Transplant-related mortality (TRM) at 30 and 100 days was 5%, 17.1% (*p*=0.086) and 27.5%, 34.2% (*p*=0.52) in the standard and intensified group, respectively. There was no difference in TRM between the 2 groups at 30 days and 100 days.

**Conclusion: **The results of the study confirm the safety of the intensified conditioning regimen. Meanwhile, it could be considered as one of the few methods available to reduce the tumor burden before HSCT for refractory malignant diseases.

## Introduction

The prognosis of allogeneic hematopoietic stem cell transplantation (HSCT) for non-remission hematological malignant disease is usually unfavorable due to the uncontrollable nature of the disease and the development of several complications ^1^^-^^6^. However, supportive therapies for allogeneic HSCT have recently been developed and to some extent, complications might be prevented with more effective drugs, anti-biotic, fungal, viral and graft-versus-host diseases (GVHD) drugs. The problem of residual disease or relapse remains unresolved. There are few therapeutic options available to address this significant problem. Basically, pre-HSCT conditioning regimens are fixed due to the limiting dose of each of the chemotherapeutic drugs and total body radiation needed to avoid organ failure, except bone marrow failure ^7^^,^^ 8^. Intensified conditioning regimens for allo- HSCT have been reported in the past. Several studies have indicated that intensified conditioning regimens did not achieve higher overall survival (OS) due to an increase in transplant-related mortality (TRM) ^9^^-^^13^. A few have reported that additional chemotherapy drugs might improve the outcome ^14^^-^^21^. Decreasing the tumor burden before HSCT, using sequential and/or additional chemotherapy to the standard conditioning regimen might be effective for advanced disease; indeed, this has been considered at our institute. On the other hand, increasing the intensity of conditioning also might lead to more patient complications such as organ failure and infection. In this report, we analyzed the safety and effects of intensified conditioning regimens. 

## MATERIALS AND METHODS

**Patients**

Patient characteristics for the analysis are summarized in Table 1. We retrospectively analyzed 81 patients with non-remission hematological malignant diseases who received allo-SCT between January 2007 and December 2011 in our institution. Forty patients used standard regimens and 41 patients received intensified regimens for SCT. Of these, 36 females and 45 were males. The average age was 51.1 years (18–68 years) for the intensified conditioning and 49.3 years (23–72 years) for the standard conditioning. The subjects were classified as 33 acute myeloid leukemia (AML), 10 myelodysplastic syndrome refractory anemia with excess blasts (MDS RAEB), 4 acute lymphoblastic leukemia (ALL), 24 malignant lymphoma (ML) and 10 adult T-cell leukemia lymphoma (ATLL).

**Table 1:** Patients characteristics

**Conditioning**	**Standard**	**Intensified**	**P-value**
Number	40	41	
Sex			
Female	20	16	0.33
Male	20	25	
Age (average)	49.3±12.7	51.1±13.1	
Age (range)	23-72	18-68	
Diagnosis			
AML	8	25	0.0001
(Blast>30%)	2	15	0.09
MDS RAEB	7	3	0.17
ALL	2	2	0.98
(Blast>30%)	1	1	1
ML	19	5	0.0004
(SD and PD)	9	2	0.78
ATLL	4	6	0.53
(SD and PD)	2	6	0.06
PS			
0	19	9	0.015
1	19	23	0.45
2	2	5	0.25
3	0	3	0.08
4	0	1	0.33
HCT-CI			
0	26	21	0.21
1	5	9	0.27
2	4	2	0.39
3	4	7	0.36
4	1	2	0.58
Infection, therapy need			
Yes	6	15	0.03
No	34	26	
T-bill			
Mild	1	4	0.18
Moderate	0	1	0.33
ALT			
Mild	0	2	0.16
Moderate	2	1	0.55
Cr			
Mild	1	3	0.32
Moderate	0	0	
Donor type			
HLA match sibling	2	4	0.42
HLA match unrelated	16	13	0.44
HLA mismatch sibling	1(haplo1)	4(haplo3)	0.18
HLA mismatch unrelated	5	6	0.78
CB	16	14	0.59
Stem cell source			
BM	22	18	0.32
PB	2	9	0.026
CB	16	14	0.59
Conditioning			
MAC	16	20	0.43
RIC	24	21	
Additional	0	22	
Sequential	0	39	
Both	0	10	

More than 30% blast cells in bone marrow was confirmed in 2 out of 8 of patients in the standard group, 15 out of 25 patients in the intensified group of AML, 1 out of 2 patients in the standard group and 1 out of 2 patients in the intensified group of ALL. Those classified as more severe than stable diseases (SD) status were: 9 out of 19 in the standard group, 2 out of 5 in the intensified group of ML, 2 out of 4 in the standard group, and all 6 in the intensified group of ATLL. Performance states (PS) were: 0 in 28 patients, 1 in 42 patients, 2 in 7 patients, 3 in 3 patients and 4 in 1 patient. Hematopoietic cell transplantation-comorbidity index (HCT-CI) ^22^ was 0 in 26, 1 in 5, 2 in 4, 3 in 4 and 4 in 1 patient of the standard regimen group and 0 in 21, 1 in 9, 2 in 2 and 3 in 7. Infectious complications were determined in 16 patients in the standard group and 23 patients in the intensified group. Liver dysfunction evaluated by T-bil was mild (>ULN to 1.5×ULN) in 4 and moderate (>1.5×ULN) in 1 patient in the standard group, mild in 4 and moderate in 1 patient in the intensified group. It was also done by ALT: mild (>ULN to 2.5×ULN) in 0, moderate (>2.5× ULN ) in 2 patients in the standard group; mild in 2, moderate in 1 patient in the intensified group. Renal dysfunction was tested by Creatinine level: mild (1.2-2mg/dl) in 1, moderate (>2mg/dl) in 0 patient in the standard group and mild in 3 patients and moderate in 0 patient in the intensified group. Definition of comorbidities was referred to HCI-CI^22^. Regarding the donor types, HLA-matched siblings were 6, HLA-matched unrelated 29, HLA-mismatched siblings 5 (haplo-identical 4), HLA-mismatched unrelated 11 and CB 30. Stem cell sources were: BM for 40 patients, CB for 30 patients and PB for 11 patients. There were 36 and 45 patients who received myeloablative conditioning (MAC) and reduced-intensity conditioning (RIC), respectively. We chose MAC regimen for patients who were under 59 years and RIC regimen for those who were over 60 years. Patients who underwent allo-SCT more than two times were excluded from the study. This study was approved by Ethics Committee of Hamanomachi Hospital.

**Standard conditioning regimens and GVHD prophylaxis**

For MAC, total body irradiation (TBI) 4 Gy×3 days + cyclophosphamide (CY) 60 mg/kg ×2 days or Busulfan (Bu) 4 mg/kg/day×4 days + CY 60 mg/kg × 2 days　were used as standard conditioning regimens. For RIC, Fludarabine (Flu) 30 mg/m2×6 days + Bu 3.2 mg/kg/day ×2–4 days + TBI 2–4Gy or Flu 25 mg/m^2^×5 days + Melphalan (Mel) 40 mg/m^2^ ×2 days +TBI 2–4Gy were used as standard conditioning regimens. MAC was used for 36 patients and RIC for 45 patients. Intensified conditioning was given to 55.6% (20/36) in the MAC group and 46.7% (21/45) in the RIC group. Prophylaxis of GVHD was done by calcineurin inhibitor (tacrolimus or cyclosporin) with short-term methotrexate (day1 10mg/m2, day3 7mg/m2 and day6 7mg/m2) or with mycophenolate mofetil (30mg/kg/day until day28 and from day29 we tried to decrease gradually and cease until day42 in the absence of active GVHD) ^23^^, ^^24^.

**Intensified conditioning regimens: sequential and/or additional chemotherapy to the standard conditioning regimens**

We defined intensified conditioning regimens as the regimens which were strengthened by adding sequential and/or additional chemotherapy to the standard conditioning regimens. The sequential conditioning regimen was defined as starting the standard conditioning regimens at a nadir before adequate hematopoietic recovery so that the numbers of white blood cells were less than 1000/μl following the most recent chemotherapy. The additional conditioning regimen was defined as added some chemotherapeutic drugs, Ara-C, VP-16, anthracyclines and monoclonal antibodies within 2 days of the standard conditioning regimen for MAC or RIC. The complete sequential regimens were given to 29 patients, the additional regimens were given to 20 patients and both were given to 8 patients. The details of the added chemotherapies are shown in Table 2a ^25^^, ^^26^.

**Statistical analysis**

Differences in Patients’ characteristics were analyzed by the Student’s t-test. Overall survival and progression -free survival were calculated from the date of stem cell transplantation using the Kaplan-Meier product-limit method, and the difference between the groups was assessed using the log-rank test. Cumulative incidence of transplant-related mortality was evaluated by the Gray test. All statistical analyses were performed using EZR version 1.30 (Saitama Medical Center, Jichii Medical University) ^27^.

## Results

**Engraftment**

Neutrophil engraftment was achieved in 85.4% patients at a median of 18.1 days following transplantation (range: 9 – 43 days) in the intensified group and in 90.0% patients at a median of 19.0 days following transplantation (range: 14 – 42 days) in the standard group. In total, 3 patients experienced primary graft failure. Of 2 patients in the intensified group, 1 had HLA antibodies and the reason for the other one was unknown. The graft of 1 patient in the standard group was also failed for unknown reason. Platelet engraftment of ≧20,000/μl was achieved in 58.5% patients in a median of 40 days (range: 12 – 270 days) in the intensified group and in 70% patients in a median of 31.6 days (range: 13 – 107 days) in the standard group.

**Acute GVHD**

The cumulative incidences of acute GVHD in grade 1–4 and 3–4 were 65.9% and 17.0% in the intensified group and 57.5% and 12.5% in the standard group, respectively (*p*=0.446, 0.568) ^28^. No statistically significant differences were observed. 

**Survival**

**Figure 1:**
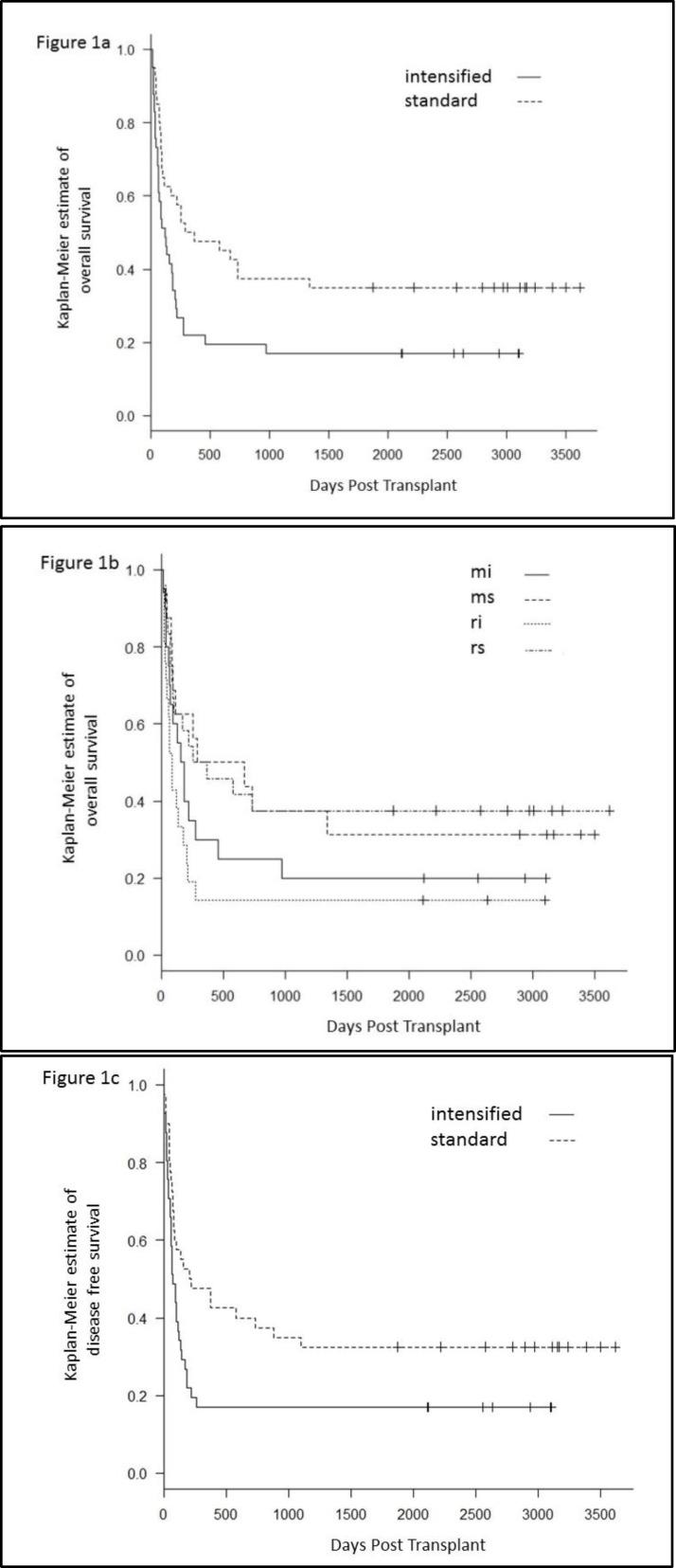
The Kaplan-Meier estimate of OS for the intensified group and the standard group (Figure 1a) and more detailed analysis for OS for each combination; RIC and standard regimens (rs), MAC and standard regimens (ms), MAC and intensified regimens (mi), RIC and intensified regimens (ri)(Figure 1b). The Kaplan-Meier estimate of progression-free survival (PFS) for the intensified group and for the standard group (Figure 1c)

The median follow-up of this cohort was 7.7 years (range, 5.0-10.0 years). The Kaplan-Meier estimate of 5-year OS was 17.1% (7.5-30.0%) for the intensified group and 35.0% (20.8-49.6%) for the standard group (*p*=0.027) (Figure 1a). Results of more detailed analysis for OS done for each combination was as follows: RIC and standard regimens (rs) 37.5% (19-56%), MAC and standard regimens (ms) 31.3% (11.4-53.6%), MAC and intensified regimens (mi) 20.0% (6.2-39.3%), RIC and intensified regimens (ri) 14.3% (3.6-32.1%) (*p*=0.099) (Figure 1b). Progression-free survival (PFS) at 5 years was 17.1% for the intensified group and 32.5% for the standard group (*p*=0.036) (Figure 1c). The results of each regimen in the sequential and/or additional conditioning regimen group are shown in Table 2b. 

**Table 2a:** Sequential and/or intensified conditioning regimen

	Intensified	
HDAC 1-3g/m2×1-2 (1-3days) based		11
VP16 5-25mg/kg (1-2days) based		8
Rituximab 375mg/m2 + ADR 50mg/body		1
Total		20
	Sequential	
HDAC 1-2g/m2×1-2 (1-3days) based		10
IDA 12mg/m2(3days) or DNR 22-45mg/m2(2-4days)		3
± LDAC 45-100mg/m2(3-7days) based		
MIT 5-7mg/m2(1-4days) +VP16 20-100mg/m2(2-5days)		6
± LDAC 70-100mg/m2(5-7days) based		
LDAC 10-100mg/m2(4-11days) based		3
GO 3mg/m2(1day) based		1
Salvage regimens for lymphoma (CHASE, Devic)(/m2) based		2
CHOP-VMMV/LSG15(/m2)(ATL regimen) based		4
Total		29
Intensified and sequential, both		8
Abbreviation: HDAC= high dose cytarabine, VP16 = etoposide, ADR = adriamycin, IDA = idamycin, DNR = daunomycin, LDAC = low dose cytarabine, MIT = mitoxantrone, GO = gemtuzumabozogamicin, (V)MMV^26^ = etoposide 35mg/m2 mitoxantrone 7mg/m2 ranimustine 50mg/m2 vindesine 2mg/m2, LSG15(ATL regimen)^25^		

**Table 2b:** Survival of the patients who received sequential and/or intensified conditioning regimen

	Patient No.	Survival	
Intensified only total ①HDAC 1-3g/m2×1-2times (1-3days) based	12 6	2 1	
②VP16 5-25mg/kg (1-2days) based	5	1	
③Rituximab 375mg/m2 + ADR 50mg/body based	1	0	
sequantial only total	21	5	
④HDAC 1-2g/m2×1-2times (1-3days) based	10	4	
⑤IDA 12mg/m2(3days) or DNR 22-45mg/m2(2-4days)	1	0	
± LDAC 45-100mg/m2(3-7days) based			
⑥MIT 5-7mg/m2(1-4days) +VP16 20-100mg/m2(2-5days)	3	0	
± LDAC 70-100mg/m2(5-7days) based			
⑦LDAC 10-100mg/m2(4-11days) based	2	0	
⑧GO 3mg/m2(1day) based	1	0	
⑨salvage regimens for lymphoma (CHASE, Devic)(/m2) based	1	0	
⑩CHOP-VMMV/LSG15(/m2)(ATL regimen) based	3	0	
Intensified and sequential, both, total	8	1	
①+⑤	2	0	
①+⑥	1	1	
①+⑦	1	0	
①+⑩	1	0	
②+⑥	2	0	
②+⑨	1	0	

**Transplant-related toxicity (TRT)**

Transplant-related toxicity, non-hematopoietic side effects of main organ dysfunction (brain, eye, lung, heart, liver, kidney, intestine and muscle), more than grade 3, was analyzed until day 100. The common terminology criteria (CTC) for adverse events, version 3.0, were used to grade the severity of side effects ^29^. Zero organ was 39%, one was 29.3%, two was 24.4%, three was 7.3% and more 

than four was 0% in the intensified group. Zero organ was 77.5%, one was 5%, two was 15%, three was 2.5% and more than four was 0% in the standard group. Documented infection, except febrile neutropenia, more than grade 3 was also analyzed until day100. 56.1% was detected in the intensified group and 40% in the standard group (Table 3).

**Table 3:** Transplant -related toxicity after stem cell transplantation Number of damaged organs (non- hematologic side effects, more than grade3)

	**Standard**	**Intensified**	**P-value**
Zero	31	16	0.0003
One	2	12	0.003
Two	6	10	0.3
Three	1	3	0.3
More than four	0	0	
Documented infection (more than grade3, except febrile neutropenia)			
	16	23	0.2

**Transplant-related mortality (TRM)**

**Figure 2:**
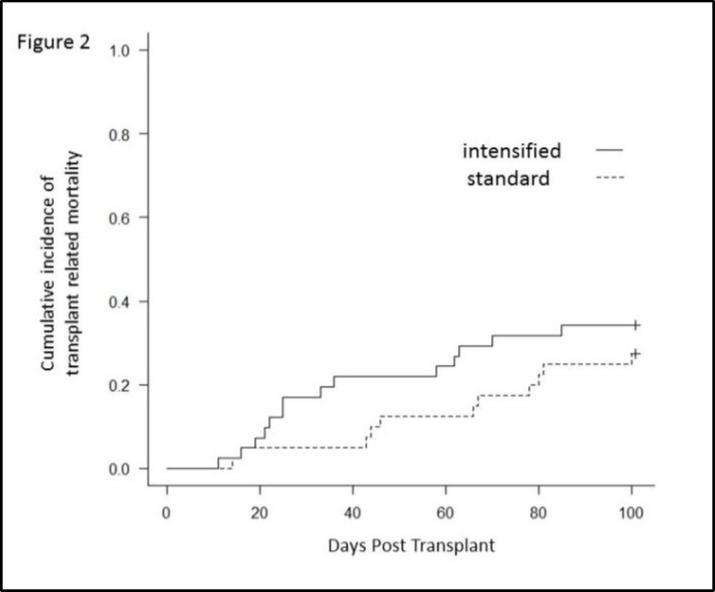
Cumulative incidence of transplant-related mortality (TRM) until day 30 and 100 days for the intensified and the standard group

Early transplant-related mortality (ETRM) until day 30 post-transplantation was 17.1% (4.7-27.8%) for the intensified group and 5.0% (0-11.5%) for the standard group (*p*=0.09). No statistically significant differences were observed. TRM at 100 days was 34.2% (17.9-47.2%) for the intensified and 27.5% (12.2-40.1%) for the standard group (*p*=0.52).There was also no statistically significant difference (Figure 2).

**Relapse**

 The cause of death is shown in Table 4. In total, 34 of 41 (82.9%) patients in the intensified group and 26 of 40 (65.0%) patients in the standard group were dead. The main causes of death were relapse or refractory disease in both groups: 15 of 41 (36.6%) in the intensified group and 12 of 40 (30.0%) patients in the standard group (*p*=0.54). Moreover, non-relapse mortality occurred in some patients who had residual or relapsed diseases. 

**Table 4:** The cause of death

	**Standard**	**Intensified**
Total	40	41
Survive	14	7
Dead	26	34
Relapse	12	15
Non relapsed	14	19
- Infection/organ failure	7	9
- GVHD/IPS	6	7
- Engraftment failure	1	0
- Others	0	3
		(Brain hemorrhage2
		Suicide 1)
		

Seven non-relapsed mortality (NRM) patients in the intensified group and 1 NRM patient in the standard group had relapse or residual disease at death. In total, 22 of 41(53.7%) patients in the intensified group and 13 of 40 (32.5%) in the standard group had residual diseases or relapses (data not shown). The disease is thought to be the biggest problem for HSCT even after the intensified regimen for those who did not achieve remission in non-remission hematological malignant diseases.

## Discussion

 In this analysis, our results suggest that the OS could not be affected by intensified conditioning for the non-remission hematological malignant diseases. However, it was at least confirmed that TRM was not also significantly different at 30 days and 100 days for both groups. The main cause of death was relapse for both groups in our study. Even after intensifying the conditioning, relapse and residual disease were the most difficult problems we encountered. In this analysis, the patients’ backgrounds were a little different. Owing to the retrospective analysis, the doctors for each patient might have tended to choose intensified conditioning if the tumor burden was great. So, the intensified group was in a ‘worse disease’ status. In fact, we could confirm that 15 of 25 (60%) AML patients in the intensified group and 2 of 8 (25%) AML patients in the standard group had more than 30% blast cells in the bone marrow prior to beginning the conditioning regimen. Furthermore, in the lymphoma and ATLL patients, the disease status in 8 of 11 (72.8%) patients in the intensified group was more advanced than SD. The same result was also obtained in 11 of 23 (47.8%) patients in the standard group. One report stated that blast cells less than 26% in the bone marrow was one of the factors that contributed to better long-term survival in patients with leukemia not experiencing remission following allo-SCT^30^. So, PFS and OS must be relatively less evaluated in the intensified conditioning group in this retrospective analysis. If the disease backgrounds are the same, they must perhaps lead to less relapse and residual disease in the intensified group after allo- SCT. There are no other options to take against the non-remission hematological diseases before the SCT conditionings other than reinforcing the conditioning. For the purpose of shrinking the tumor burden, intensified conditioning is one of the meaningful options that can guarantee safety. Toxicity and safety of conditioning regimens were determined before SCT in each situation^7^^,^^8^. However, a few reports have stated that the intensified conditioning is better than the standard one^14^^-^^21^. Some authors also mention that different results were seen by the different doses and various combinations of chemotherapeutic drugs used in conditioning regimen^2^^, ^^16^^, ^^17^^, ^^31^^, ^^32^. Using novel conditionings in combination with new drugs have been developed showing better insight^33^^, ^^34^. In our analysis, ETRM was 17.1% for the intensified group and 5.0% for the standard group (*p*=0.09), which was not significantly different. TRM at 100 days was 34.2% for the intensified and 27.5% for the standard group (*p*=0.52). Meanwhile, no statistically significant difference was found in the non-remission cases. According to the results, we can try to modify the conditioning to get a better outcome by doing sequential and/or intensified chemotherapy, carefully considering the age, PS, organ function, etc. In general, to control the relapse and　refractory hematological malignant diseases following SCT, we can employ several methods: intensifying conditions, modifying the blood concentration of immunosuppressive drugs ^20^, inducing graft- versus- leukemia/lymphoma (GVLL) effects, using donor lymphocyte infusion (DLI) ^35^^,^^36^, selecting haploidentical siblings for donors ^37^^,^^38^, minimal residual disease and chimerism monitoring for pre-emptive administration^35^^,^^39^, etc. Recently, supportive therapy has progressed and, in cases of relapse, PS and organ function are often preserved and retransplantation could be considered ^40^^,^^41^. Regarding the sources of SCT, the use of cord blood or haploidentical sibling peripheral blood stem cells, which can be prepared easily and quickly, is gradually increasing and, by repeated SCT experiences, its use is becoming more familiar ^37^^,^^38^^,^^42^. Inducing GVHD/GVLL was also the choice for relapse disease by decreasing and ceasing the immunosuppressive drugs, tacrolimus, cyclosporine and DLI ^20^^,^^35^^,^^36^. Too much damage to the patient caused by conditioning regimen with cytotoxic drugs and radiation would narrow down the choices for relapse or residual disease. But, the outcome after relapse or residual disease after SCT are not usually favorable ^35^^,^^36^^,^^39^^-^^41^.Cure by means of single allo-SCT might be a simpler and better approach. Totally, intensified conditioning could be considered in cases of non-remission disease before HSCT.

## CONCLUSION

Our study indicates that TRM of intensified regimens was not significantly different from that of standard regimens at 30 days and 100 days. The results also suggest that the intensified conditioning regimens can be employed just before HSCT to improve survival in patients with non-remission disease.
